# Altered Short-Term Synaptic Plasticity in Mice Lacking the Metabotropic Glutamate Receptor mGlu7

**DOI:** 10.1100/tsw.2002.146

**Published:** 2002-03-15

**Authors:** Trevor J. Bushell, Gilles Sansig, Valerie J. Collett, Herman van der Putten, Graham L. Collingridge

**Affiliations:** MRC Centre for Synaptic Plasticity, Department of Anatomy, The School of Medical Sciences, University of Bristol, University Walk, Bristol, BS8 1TD, UK; Biophysics Section, Blackett Laboratory, Department of Biological Sciences, Imperial College of Science, Technology and Medicine, Prince Consort Road, London, SW7 2BW, UK; Nervous System Department, Novartis Pharma AG, CH-4002 Basel, Switzerland

**Keywords:** mGluR7, mGlu7 receptor, metabotropic glutamate receptor, hippocampus, hippocampal, CA1, Schaffer collateral-commissural pathway, synaptic transmission, synaptic plasticity, long-term potentiation, short-term potentiation, LTP, STP, knockout, homologous recombination

## Abstract

Eight subtypes of metabotropic glutamate (mGlu) receptors have been identified of which two, mGlu5 and mGlu7, are highly expressed at synapses made between CA3 and CA1 pyramidal neurons in the hippocampus. This input, the Schaffer collateral-commissural pathway, displays robust long-term potentiation (LTP), a process believed to utilise molecular mechanisms that are key processes involved in the synaptic basis of learning and memory. To investigate the possible function in LTP of mGlu7 receptors, a subtype for which no specific antagonists exist, we generated a mouse lacking this receptor, by homologous recombination. We found that LTP could be induced in mGlu7-/- mice and that once the potentiation had reached a stable level there was no difference in the magnitude of LTP between mGlu7-/- mice and their littermate controls. However, the initial decremental phase of LTP, known as short-term potentiation (STP), was greatly attenuated in the mGlu7-/- mouse. In addition, there was less frequency facilitation during, and less post-tetanic potentiation following, a high frequency train in the mGlu7-/- mouse. These results show that the absence of mGlu7 receptors results in alterations in short-term synaptic plasticity in the hippocampus.

## INTRODUCTION

It is widely believed that synaptic plasticity is the primary mechanism utilised by the brain to enable the acquisition and storage of information. Most information concerning the molecular and cellular basis of synaptic plasticity has been derived from studies of long-term potentiation (LTP) in the hippocampus[1,2,3]. LTP is an increase in synaptic efficiency that is induced within a minute and lasts for many hours or days. In most hippocampal pathways, such as the Schaffer collateral-commissural inputs from CA3 to CA1 neurons, the induction of LTP requires the transient synaptic activation of N-methyl-D-aspartate (NMDA) receptors[1,2,3]. This results in a persistent increase in the amplitude of α-amino-3-hydrox-5-methyl-4-isoxazolepropionate (AMPA) and NMDA receptor–mediated synaptic transmission. The mechanisms that are involved following the activation of NMDA receptors, to result in the long-lasting increase in synaptic efficiency, are not fully understood but are the subject of intense investigation[4,5,6,7]. In addition to NMDA receptors, there is considerable evidence that mGlu receptors are also involved in the induction of LTP, under certain circumstances. The most direct evidence comes from studies using the mGlu receptor antagonist α-methyl-4-carboxyphenylglycine (MCPG), which has been shown to block the induction of LTP under some[8,9,10] but not all[10,11,12] conditions.

MCPG is a "broad spectrum" antagonist since it acts on several (mGlu1, mGlu2, mGlu3, mGlu5, and mGlu8) of the mGlu receptors subtypes (mGlu1-mGlu8) that have been identified by cloning[13]. Since the discovery of MCPG[14], there have been many more mGlu receptor antagonists developed, including some that are specific for a single mGlu receptor subtype[13](see also URL: http://www.bris.ac.uk/Depts/Synaptic/info/pharmacology/mGlu.html). However, potent subtype-specific mGlu receptor antagonists are still lacking for many of the mGlu receptor subtypes, in particular mGlu7. An alternative strategy to investigate the functional roles of mGlu receptors is to use homologous recombination gene targeting. This has the advantage of a highly specific elimination of the receptor of interest but the disadvantage of potential developmental compensation. Most of the members of the mGlu receptor family have been "knocked out" and the effects of the deletion on synaptic function investigated[15,16,17,18,19](see also URL: http://www.bris.ac.uk/Depts/Synaptic/info/tools.html). At CA1 synapses the two mGlu receptors that are expressed at the highest levels are mGlu5 and mGlu7[20,21,22,23]. Thus, these are the two most likely mGlu receptor subtypes to be involved in the induction of LTP at these synapses. Deletion of mGlu5 receptors prevents the induction of LTP of NMDA receptor–mediated synaptic transmission without affecting LTP of AMPA receptor–mediated synaptic transmission[24]. However, the function of mGlu7 receptors in synaptic plasticity at CA1 synapses is unknown.

We have generated an mGlu7 knockout[25]. Previously we have reported that this knockout results in deficits in taste aversion and fear responses[26] and a predisposition to epilepsy[25]. In the present study, we have compared LTP at CA1 synapses in this knockout. We find that LTP can be induced normally in the mGlu7 knockout. However, there is an impairment of the earliest phase of LTP, known as short-term potentiation (STP), and an impairment in both frequency facilitation and post-tetanic potentiation.

## METHODS

Mice were generated by homologous recombination gene targeting as described previously[25]. Slices (400 μm thick) were prepared from the hippocampi of 5–10 week old mutant mice and littermate wildtypes using standard procedures. The slices were submerged in a medium which comprised (mM): NaCl 124; KCl 3; $NaHCO_3$ 26; $NaH_2PO_4$ 1.4; $MgSO_4$ 1; $CaCl_2$ 2; D-glucose 10; (bubbled with 95% $O_2$/5% $CO_2$; pH 7.4), and were perfused at a rate of approximately 4 ml

$min^{-1}$ (29–31°C). Extracellular recordings were obtained from stratum radiatum of area CA1 in response to low frequency (0.033 Hz) stimulation of the Schaffer collateral-commissural pathway. Measurements of the slope of the field EPSP were used throughout. Tetanic stimulation (100 Hz, 1 s, test intensity) was delivered at the times indicated by arrows (Fig. 1a,b). Data acquisition and analysis was performed using the "LTP" program (URL: http://www.ltp-program.com/). For each protocol, one slice was used per animal, hence n values give the number of slices/mice used. Statistical significance was determined using a repeated measures ANOVA and unpaired Students t-tests. * Denotes $p < 0.05$. Animals were genotyped by PCR and presented to the experimenter in a randomised and blind manner.

## RESULTS

We have reported previously that there are no obvious differences in basal synaptic properties in the CA1 region of hippocampal slices[25]. We applied a single tetanus (100 Hz, 1 s, test intensity) to mGlu7 receptor knockouts (n = 12) and littermate controls (n = 14). The level of LTP, measured at 60 min following tetanic stimulation, was not significantly different between groups (Fig. 1). However, the initial phase of potentiation, which is commonly referred to as STP, was markedly reduced in the mutant mice. For example, the initial measure of potentiation, obtained between 0.5 and 2 min, for the respective groups was 89 ± 15% and 163 ± 21% ($p <$ 0.01) and the differences remained statistically different for 16 min following tetanic stimulation (Fig. 1).

To determine whether there were differences in the synaptic responses during, and shortly following, high frequency transmission in the two groups of mice a second set of experiments were performed. In these experiments, LTP was firstly saturated by applying multiple tetani, so that high frequency responses could be analysed without the complications of the induction of LTP or the presence of pharmacological antagonists. Twenty shocks delivered at 100 Hz were followed by a test pulse delivered either 50 or 500 ms later. During the high frequency train, the size of individual AMPA receptor–mediated EPSPs increased and then decreased in both groups. However, from approximately 30–40 ms into the train, the EPSPs were smaller, relative to the first EPSP of the train, in the mutant mice (Fig. 2). The post-tetanic response, assessed as the ratio of the test EPSP to the first EPSP in the train, was also reduced in the mutant mice (Fig. 2); the ratios obtained, using respective interpulse intervals of 50 and 500 ms, were 2.16 ± 0.01 and 0.88 ± 0.07 for wild-type (n = 8) and 1.43 ± 0.11and 0.65 ± 0.05 for mutant (n = 8) mice ($p < 0.05$). In addition to changes in EPSPs, there was also a reduction in the size of the summated IPSPs during and following the train in mutant mice (Fig. 2).

## DISCUSSION

In the present study we have identified differences in the response to high frequency stimulation between mGlu7 knockout mice and their wildtype littermates. The absence of mGlu7 receptors was associated with a depression in synaptic transmission during and following the high frequency train and a reduction in STP.

Although these effects can be attributed to the absence of mGlu7 receptors it is not possible to determine whether it is the acute loss of these receptors or a consequence of their absence during development that leads to these alterations in synaptic transmission and plasticity. This will require the development of conditional mGlu7 receptor knockout mice or specific mGlu7 receptor antagonists. However, some discussion of this issue is warranted. Although there are currently no selective mGlu7 receptor antagonists, compounds that inhibit mGlu7 receptors together with other mGlu receptor subtypes do exist. The most potent of these is LY341495, with

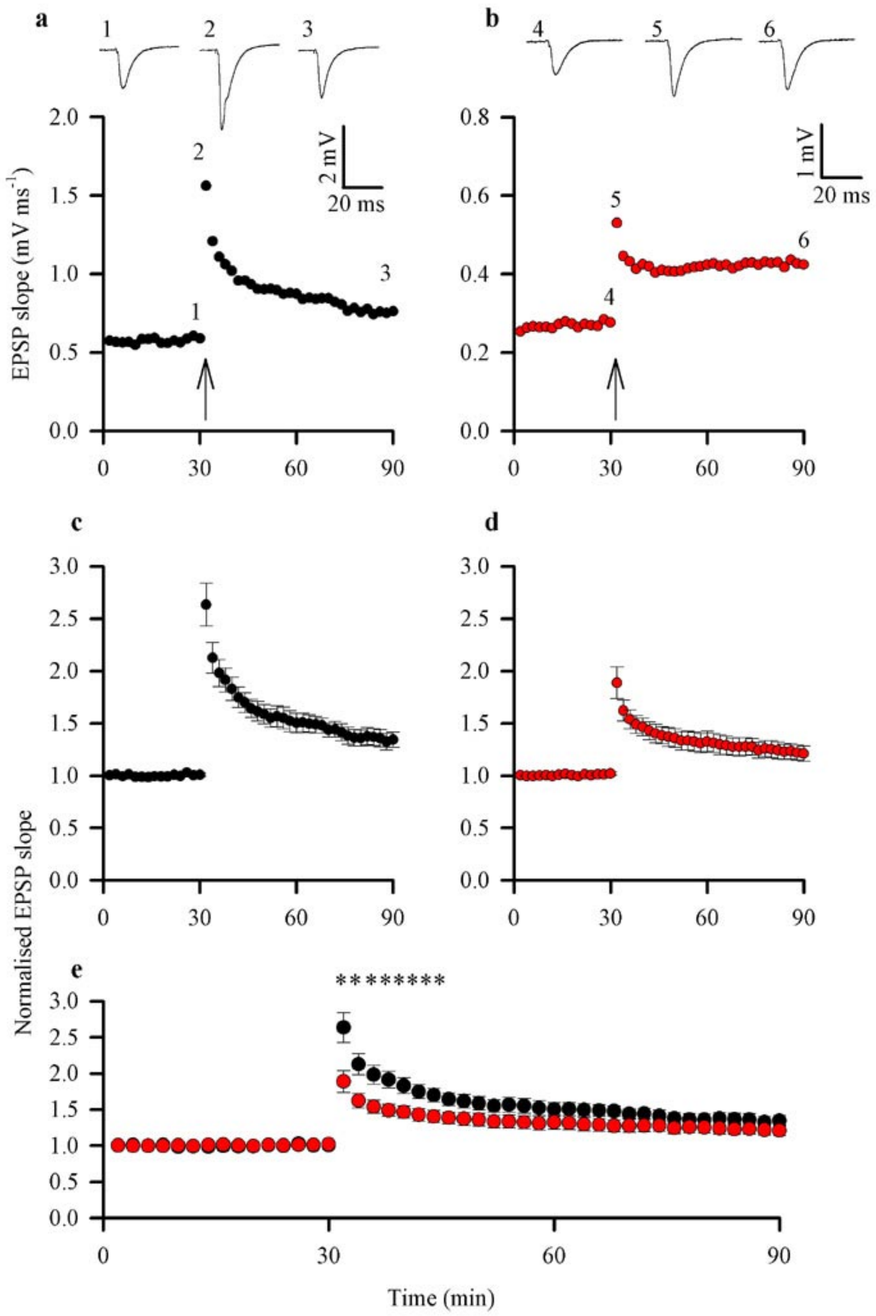

**FIGURE 1.** STP is reduced in mGlu7 knockout mice. Representative examples of individual experiments are shown for wildtype (a) and mGlu7 knockout (b) mice. The corresponding pooled data for 14 slices from wildtype (c) and 12 slices from mGlu7 knockout (d) mice are superimposed (e) to illustrate the extent of the deficit in STP.

an $IC_{50}$ of approximately 1 μM[27] at mGlu7 receptors. LY341495 (100 μM) does not affect STP or LTP at CA1 synapses[28]. This suggests that either the effects observed with the mGlu7 knockout are developmental in origin or that the effects of LY341495 on mGlu7 receptors is compensated for by the simultaneous antagonism of other mGlu receptor subtypes.

If the alterations in the mGlu7 receptor knockout are due to the acute loss of receptor (i.e., would be mimicked by a specific mGluR receptor antagonist) the implication is that mGlu7

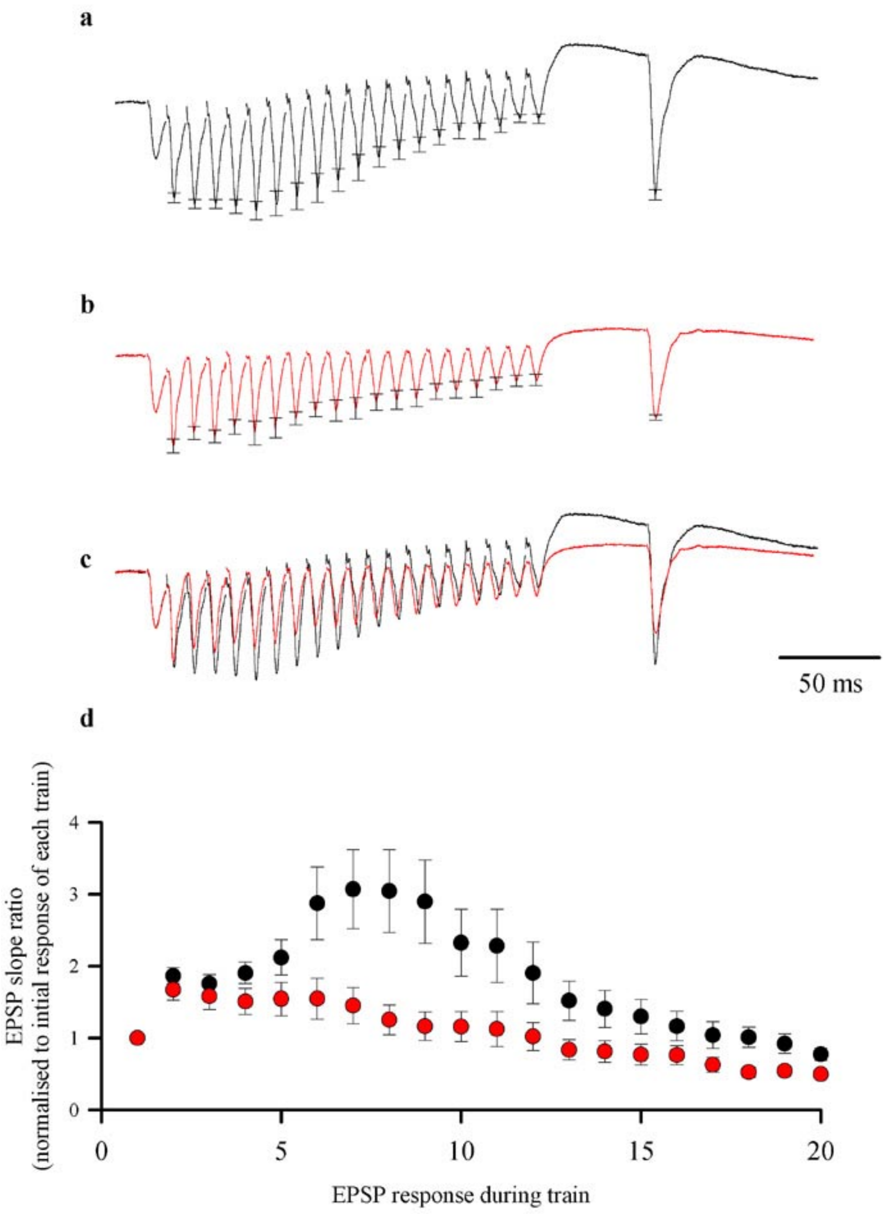

**FIGURE 2.** High frequency synaptic transmission is reduced in mGlu7 knockout mice. Pooled traces illustrating reduced AMPA receptor–mediated EPSPs during the train and in response to the test pulse for 8 slices from wildtype (a) and 8 slices from mGlu7 knockout (b) mice. The ratio of each EPSP in relation to the initial response of each train is shown superimposed (c).

receptors function as facilitatory autoreceptors at these synapses. Their well-established presynaptic location[21,29] close to release sites[21], together with their low affinity for L-glutamate, makes them ideally placed to function as autoreceptors. How their activation could lead to enhanced L-glutamate release is a matter for speculation. One possibility, based on work using retinal horizontal cells, is via stimulation of guanylyl cyclase, elevation of cGMP and inhibition of potassium channels[30].

The alterations in the activation of AMPA and GABA receptors during the high frequency train means that it is likely that the level of activation of NMDA receptors was also different[1]. Presumably, in both sets of mice there was sufficient synaptic activation of NMDA receptors for

LTP to be induced normally. However it is possible that with different patterns of activation, such as those closer to the threshold for LTP induction, differences in LTP might be observed.

An interesting observation, under the conditions of the present experiments, is that the stable level of LTP generated was not directly related to the initial level of STP. Treatments that affect tetanus-induced synaptic potentiation generally either depress STP and LTP in parallel or, like many protein kinase inhibitors and targeted deletions of protein kinase genes, selectively block LTP[31,32,33,34,35,36]. This has led to the belief that STP and LTP are serial processes where STP is required for, but does not necessarily lead to, LTP[37,38]. The present results suggest, however, that the level of STP can be regulated independently of the level of stable LTP.

In conclusion, our experiments suggest that mGlu7 receptors influence high frequency synaptic transmission and short-term synaptic plasticity in the hippocampus.

## ACKNOWLEDGEMENTS

Supported by the MRC.
